# Supplementary material for: Community detection for directed networks revisited using bimodularity
Source: Proc Natl Acad Sci U S A. 2025 Aug 25;122(35):e2500571122. doi: 10.1073/pnas.2500571122 (PMC12415231; doi:10.1073/pnas.2500571122)
Supplement: Supplementary file 1 — Appendix 01 (PDF) [file pnas.2500571122.sapp.pdf]

Supporting Information for

Community detection for directed networks revisited using bimodularity

Alexandre Cionca, Chun Hei Michael Chan and Dimitri Van De Ville

Dimitri Van De Ville
E-mail: dimitri.vandeville@epfl.ch

This PDF file includes:

Figs. S1 to S10
Legend for Dataset S1
SI References

Other supporting materials for this manuscript include the following:

Dataset S1

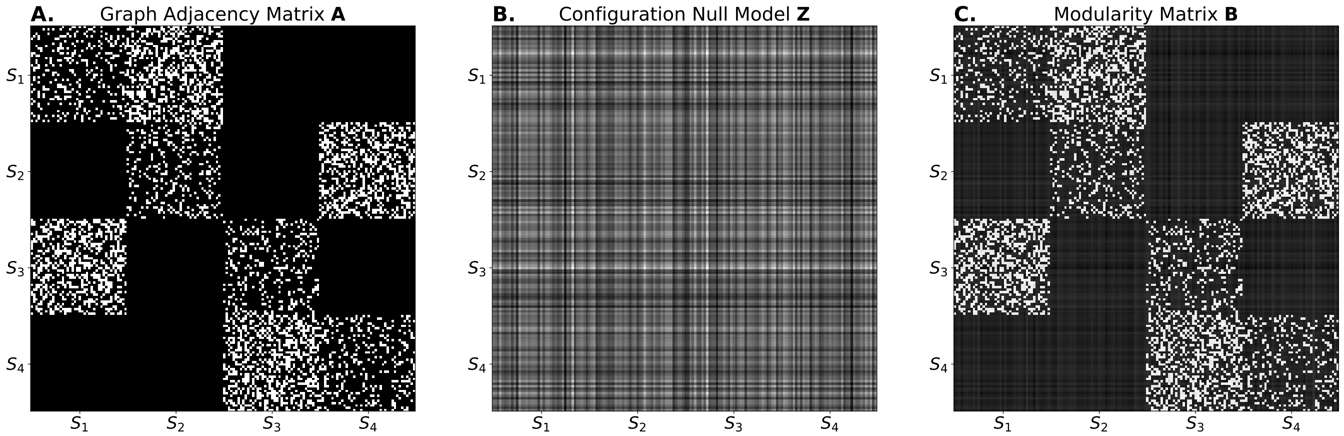

Fig. S1. Adjacency, null model and modularity matrices for the canonical block cycle graph. (A) Adjacency matrix $\mathbf{A}$ of a stochastic block graph of 200 nodes (50 per community) with the structure of A. (B) Configuration null model with the same degree distribution as $\mathbf{A}$. (C) Modularity matrix $\mathbf{B}$ of $\mathbf{A}$.

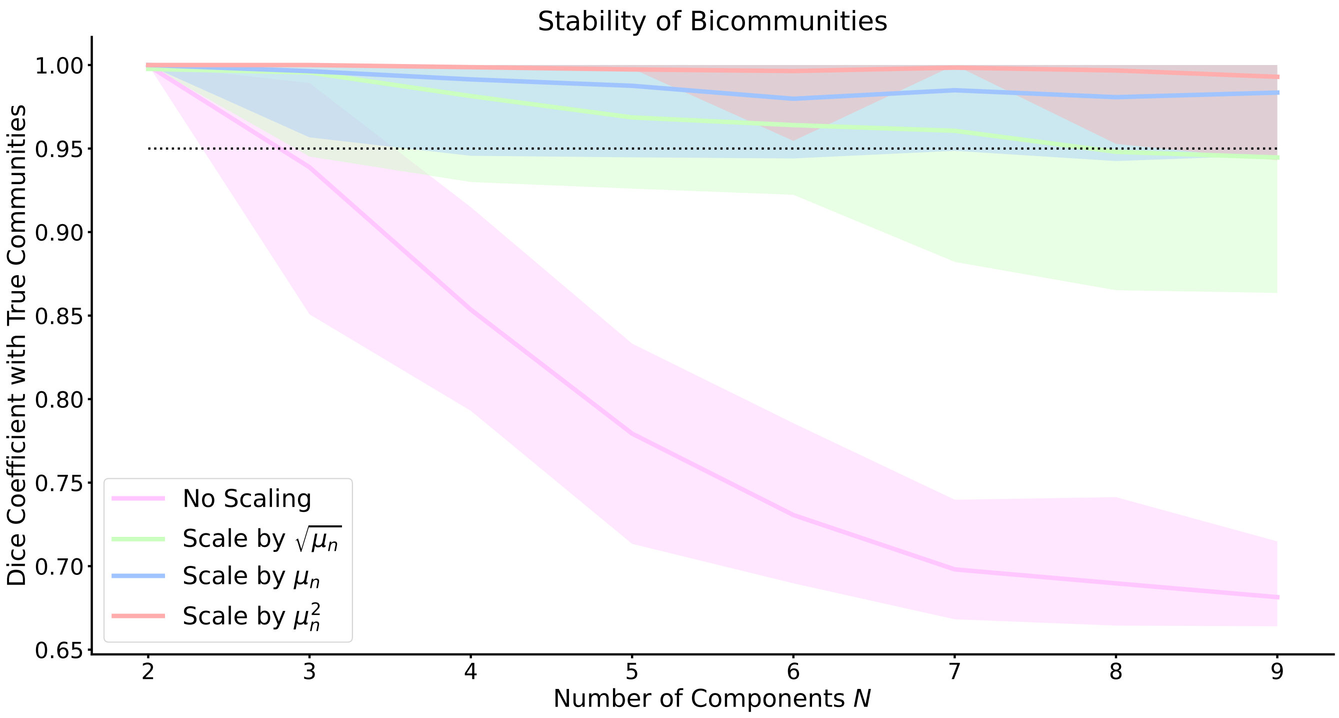

Fig. S2. Stability of bicommunity detection in the canonical block cycle graph as measured with the Dice coefficient. The stability is computed as a function of the number of components of the singular value decomposition (SVD – horizontal axis) and for different scaling of the components (colors). The filled area represent the 5 and 95 percentile out of 100 trials. We observe that the scaling of components (even in the case of $\sqrt{\mu_n}$) improves the stability of the detection and that the μ_n scaling ensures a similarity higher than 95%.

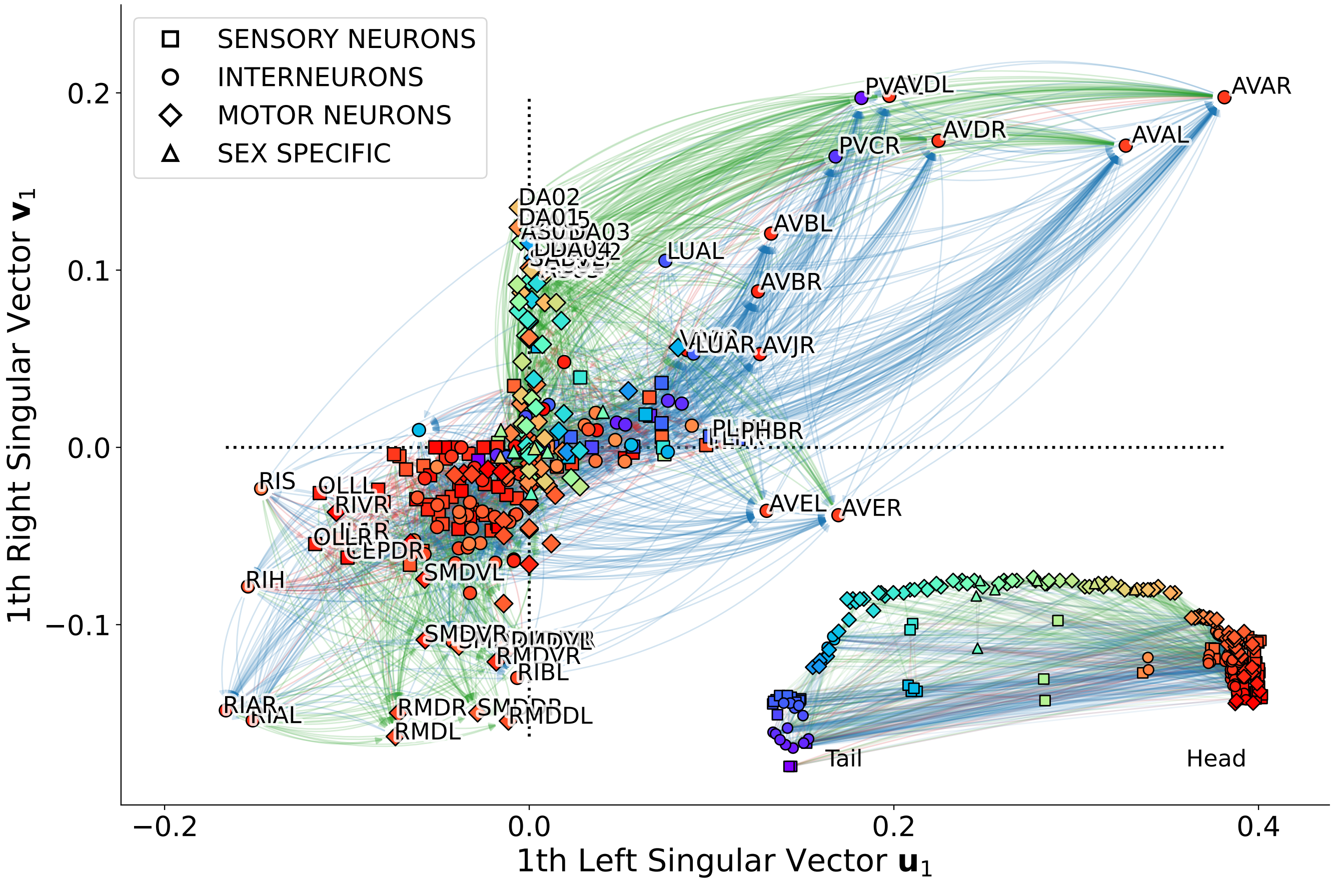

Fig. S3. Bimodular embedding of the *C. elegans* wiring network with physical location of neurons. This shows the projection of the graph nodes (neurons) onto the left ($\mathbf{u}_1$, horizontal axis) and right ($\mathbf{v}_1$, vertical axis) singular vectors of the bimodular component associated with the largest singular value μ_1. Node color indicate the physical location of the neuron in the worm's body from the head (read) to the tail (blue). Shape of nodes indicate the type of the neurons (sensory: square; interneuron: circle; or motor: diamond). Edge colors indicate the type of the target node with red for sensory, bleu for inter- and green for motor neurons. Neuron labels are shown for graph nodes that are projected far from the origin $(0, 0)$ of the axes. The legend in the bottom right shows each neuron at their physical location in the worm's body.

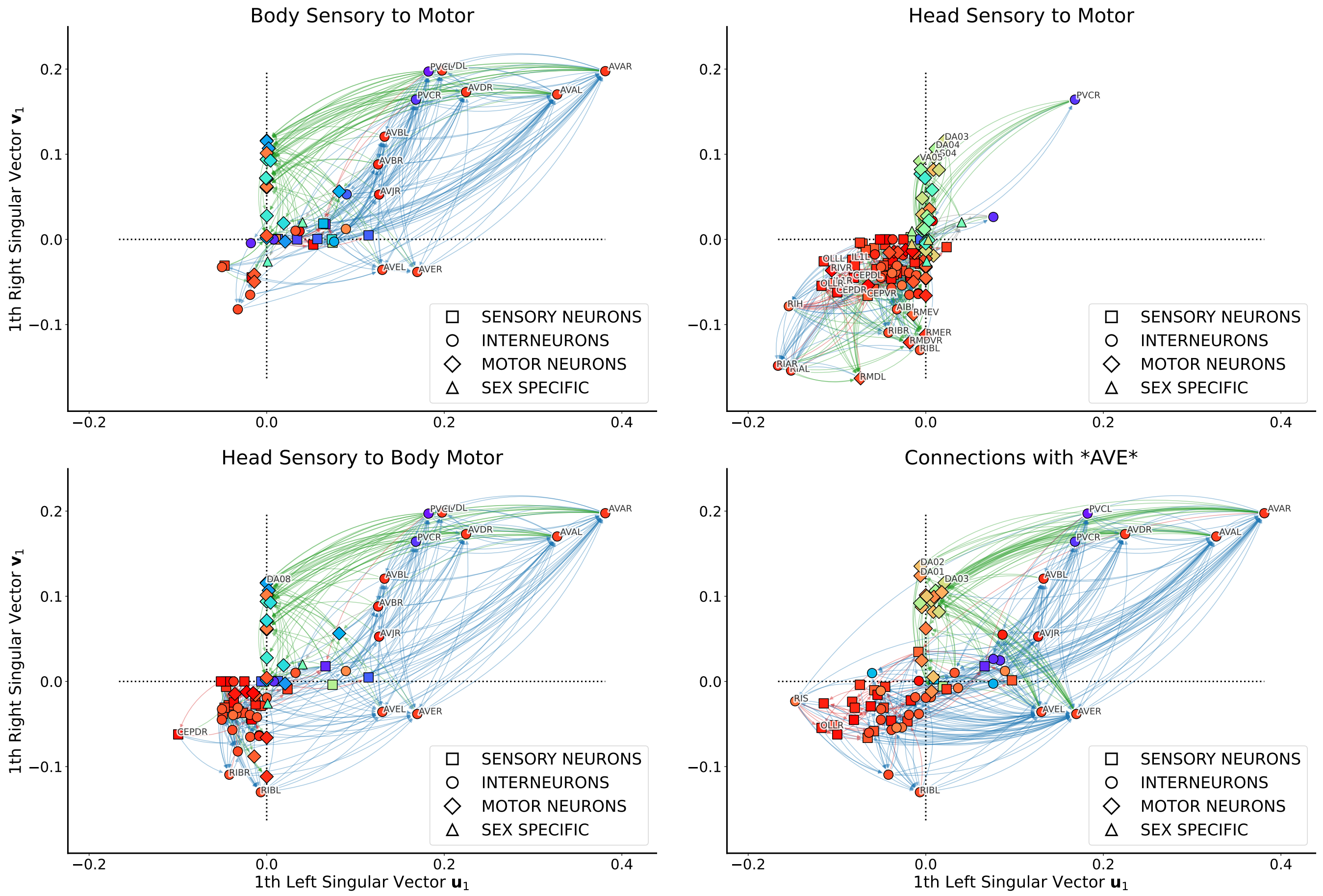

Fig. S4. Communication pathways of the *C. elegans* wiring network projected on the first bimodular embedding. Specific pathways are highlighted by discarding all nodes and connections that are not connected to a source (zero in-degree $k_{\text{in}} = 0$) or sink (zero out-degree $k_{\text{out}} = 0$) node. Sensory to motor communication in the worm's body (top left) is highlighted by considering communication with source nodes on the right half ($\mathbf{u}_1 > 0$) and sink nodes on the upper half ($\mathbf{v}_1 > 0$). The head sensory to motor pathway (top right) considers communication with source nodes on the left half ($\mathbf{u}_1 < 0$) and sink nodes on the lower half ($\mathbf{v}_1 < 0$). Finally, head sensory to body motor communication (bottom left) highlights neurons that are linked with left sources and upper sinks. This pathways shares similarities with the bottom right scheme that shows all neurons connected to the *AVE* interneurons that appear to bridge head sensory inputs and body motor processes. Node color indicate the physical location of the neuron in the worm's body from the head (read) to the tail (blue). Shape of nodes indicate the type of the neurons (sensory: square; interneuron: circle; or motor: diamond). Edge colors indicate the type of the target node with red for sensory, bleu for inter- and green for motor neurons. Neuron labels are shown for graph nodes that are projected far from the origin $(0, 0)$ of the axes.

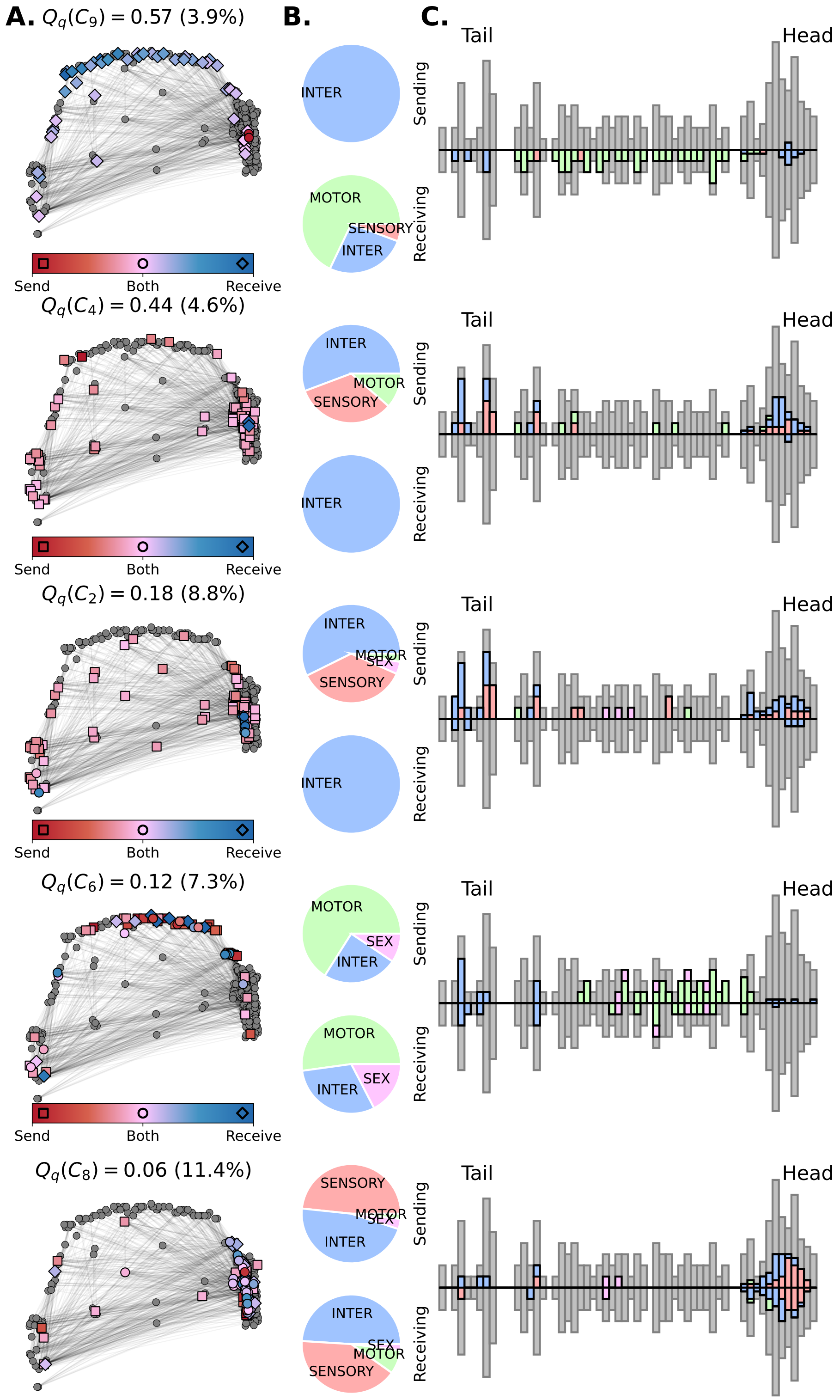

Fig. S5. Bicommunity of the *C. elegans* extracted from the $k = 9$ first bimodularity embeddings (components of the SVD). Only the clusters with top 5 highest bimodularity index (out of 9) are displayed. A. The sending and receiving parts of each bicommunity are highlighted on the spatial location of neurons. Markers indicate whether a neuron belongs to the sending (red square), the receiving (blue diamond) or both (pink circle) parts of a bicommunity. The colors represent the difference between the proportion of edges in the sending and in the receiving partitions with red and blue for nodes that tend to send and receive more respectively. B. The distribution of neuron types (sensory, inter, motor and sex-specific neurons) in each sending or receiving community is summarized in pie charts. C. The spatial distribution of bicommunities along the tail to head axis is detailed in the histograms as a proportion of the total number of neurons (gray bars). The sending and receiving patterns are separated in the upper and lower bars respectively. Colors indicate the type of neuron following the color scheme of the pie charts. The body and tail histograms are made bigger (twice their original size/number of neurons) for visualization purpose. We observe that the first 4 components are coherent with the 3 bicommunities presented in the main text (with $k = 5$). They first highlight communication from sensory neurons of the head and body to key interneurons *AVE* (rows 2 and 3), then show communication from these same interneurons to motor neurons of the body (row 1). Finally, interconnections between motor neurons of the body and interneurons of the tail are highlighted in the 4th row.

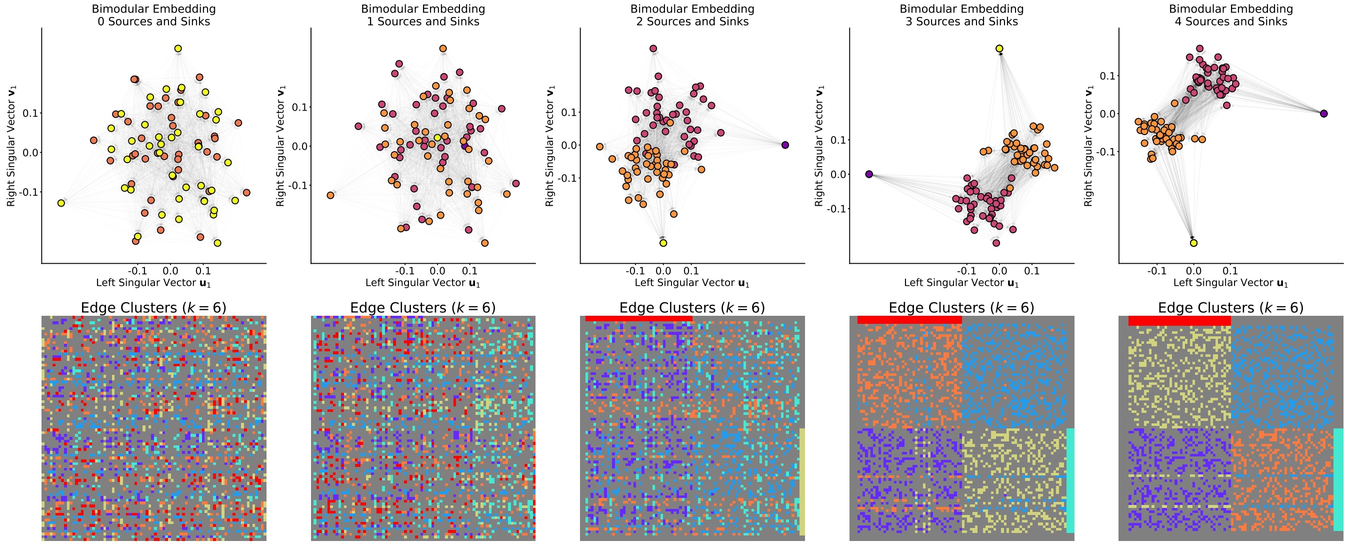

Fig. S6. Canonical example of two sets of nodes (red and orange) that are randomly interconnected with the addition of source (purple) and sink (yellow) nodes with high degree imbalance and that connect to either of the node sets. The first row shows the first bimodular embedding of the graph for each number of source and sink node (from 0 to 4). The second row shows the edge clusters as obtained from the bicommunity detection scheme for $k = 6$ clusters. We observe that 2 added sources/sinks are enough to partially recover the encoded structure and to separate the two sets of nodes. The sources and sinks also are well separated. The two sets are perfectly separated at 3 added nodes and 4 are required to totally recover the 6 clusters of edges.

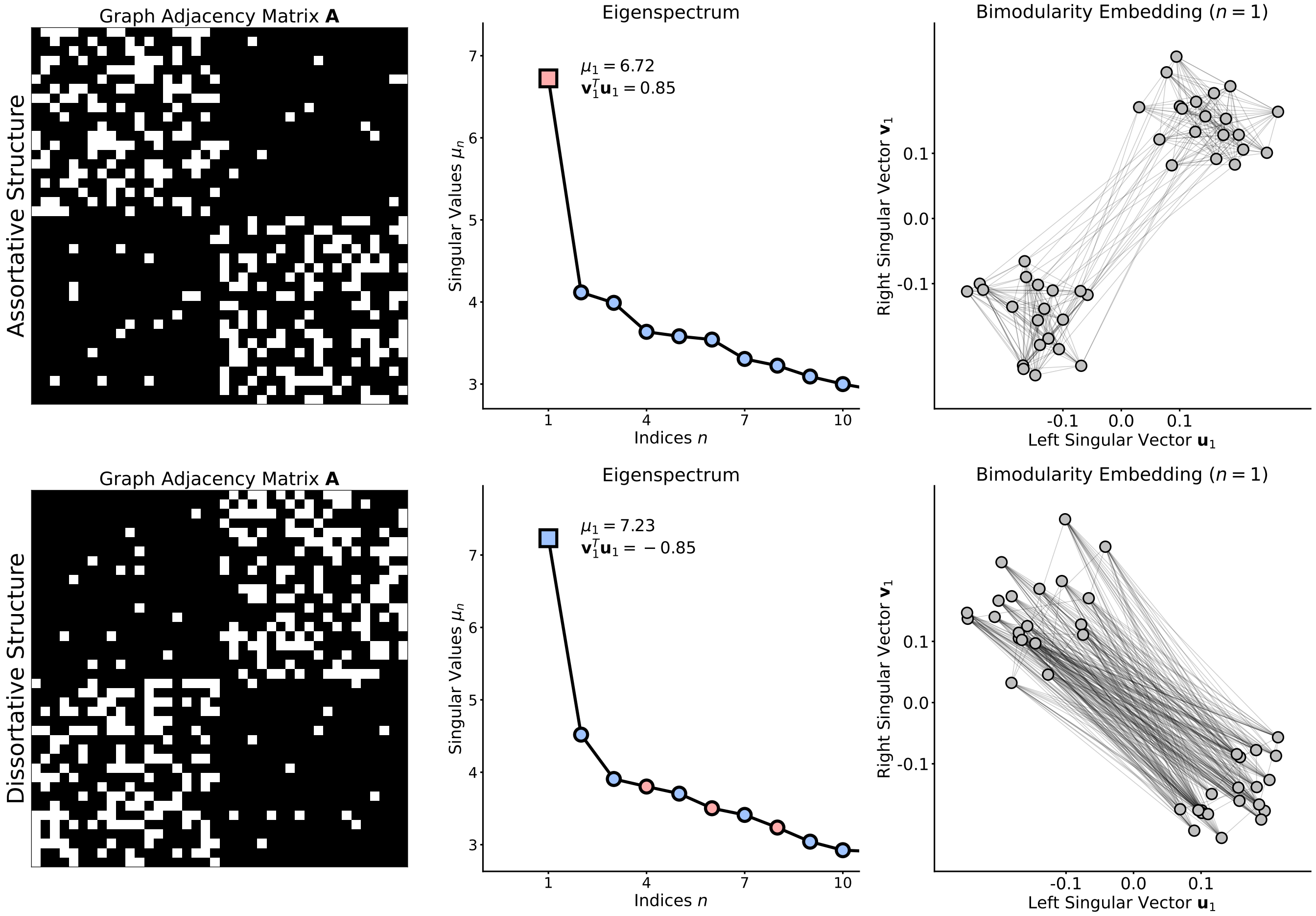

Fig. S7. Canonical example of assortative (top) and dissortative (bottom) community structure. While the singular values (middle) have a similar spectrum, we observe a clear difference in the sign convention with $\mathbf{v}_1^T\mathbf{u}_1 = 0.85$ in the assortative and $\mathbf{v}_1^T\mathbf{u}_1 = -0.85$ in the dissortative case. The bimodular embeddings (right) show a clear community structure in the assortative graph and a bipartite structure in the dissortative graph. We note that the dissortative embedding separates well the graph nodes in the $x = -x$ diagonal as opposed to the $x = x$ diagonal in the assortative condition.

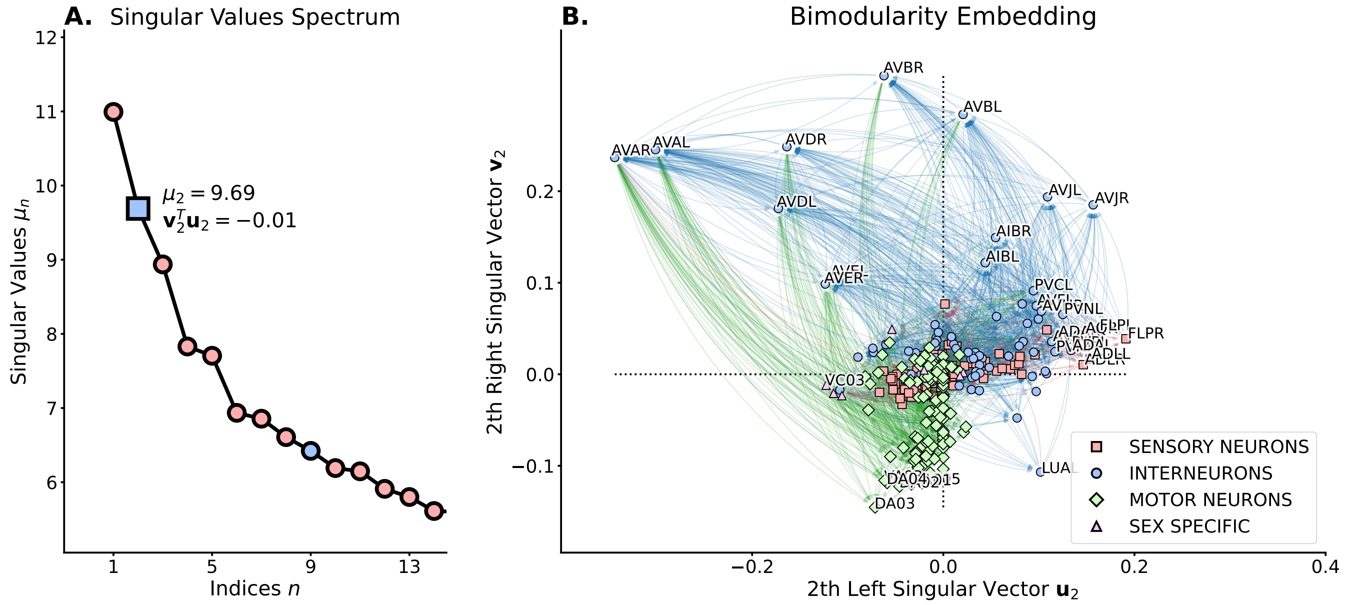

Fig. S8. Bimodular embedding of the *C. elegans* wiring network for the 2nd component of the Singular value decomponsition (SVD). (A) Spectrum of the singular values of the SVD. Colors indicate the sign of the singular value associated to assortative (red, $\mu > 0$) or dissortative (blue, $\mu < 0$) community structures. We observe that while μ_2 has a high value, this component describes a dissortative structure ($\mathbf{v}_2^T\mathbf{u}_2 < 0$). (B) Projection of the graph nodes (neurons) onto the left ($\mathbf{u}_2$, horizontal axis) and right ($\mathbf{v}_2$, vertical axis) singular vectors of the bimodular component associated with the largest singular value (μ_2). Colors and shape of nodes indicate the type of the neurons (sensory: red square; interneuron: blue circle; or motor: green diamond). Edge colors indicate the type of the target node with the same color code as for the neuron types. Neuron labels are shown for graph nodes that are projected far from the origin $(0, 0)$ of the axes.

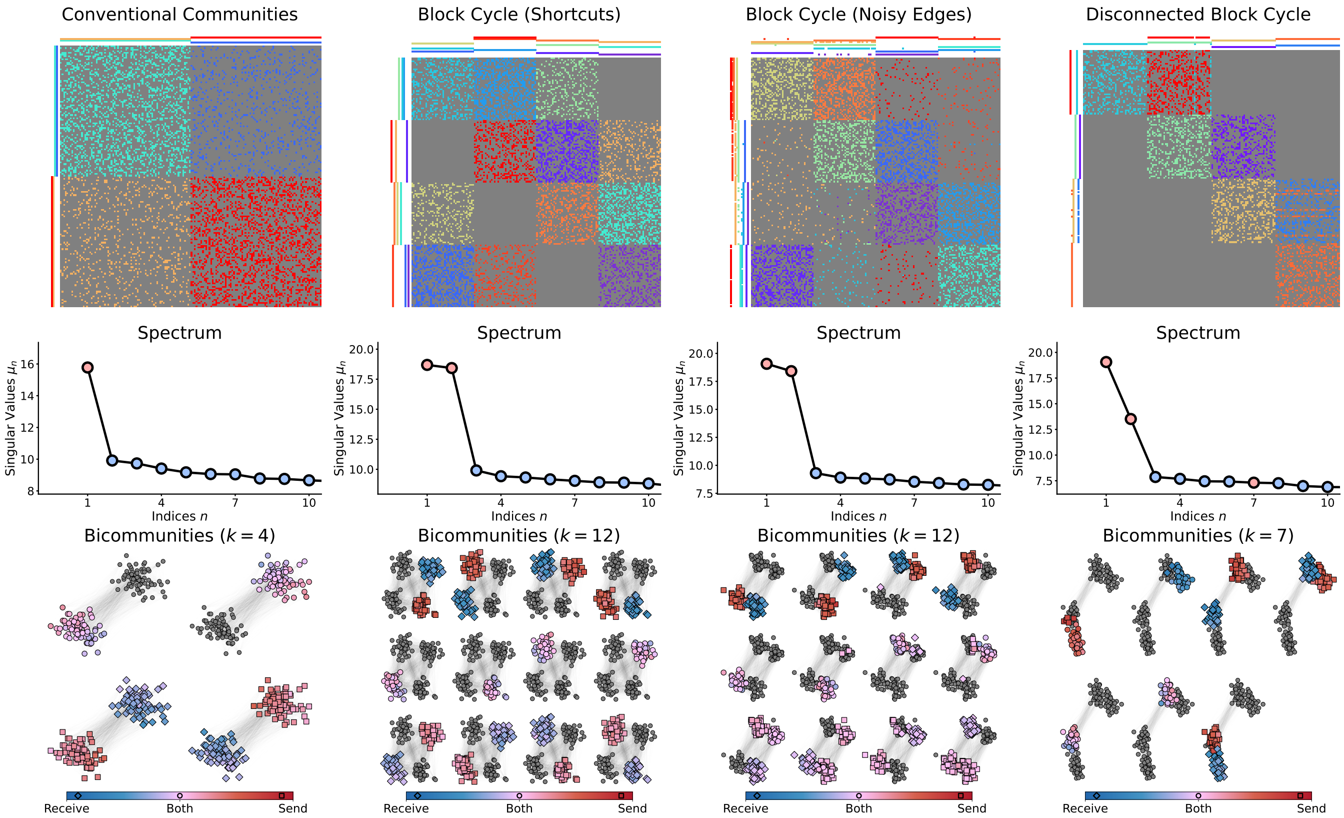

Fig. S9. Benchmarking of the bimodularity embedding and bicommunity detection for 4 types of graphs (each column): Conventional community with two sets of nodes that have directed connections within (density 0.6) and between (density 0.24) them; Block cycle graph with bidirectional shortcuts between blocks that are not connected; Block cycle with random edges between nodes; and block cycle with a missing connection between the last and first blocks. The first row shows the adjacency matrix with color corresponding to the edge clusters. In all cases, the encoded structure is recovered and all sets of edges are properly captured in their respective clusters.

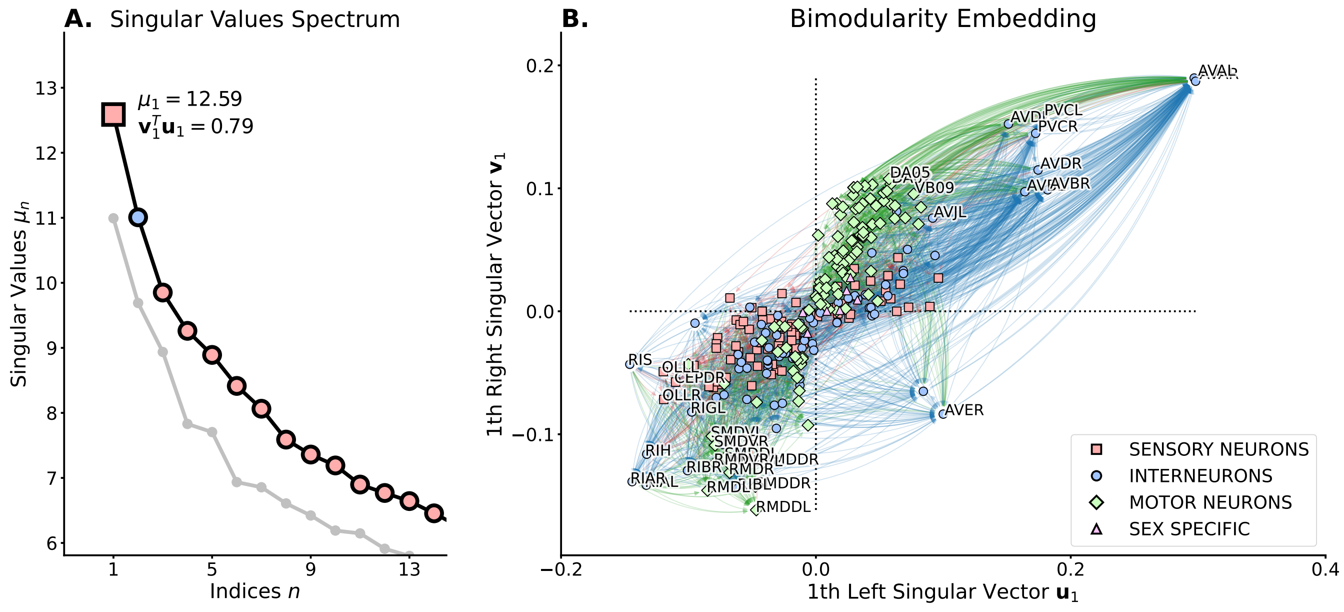

Fig. S10. Bimodular embedding of the *C. elegans* wiring network with added undirected electrical gap junction network. (A) Spectrum of the singular values of the SVD. Colors indicate the sign of the singular value associated to assortative (red, $\mu > 0$) or dissortative (blue, $\mu < 0$) community structures. (B) Projection of the graph nodes (neurons) onto the left ($\mathbf{u}_2$, horizontal axis) and right ($\mathbf{v}_2$, vertical axis) singular vectors of the bimodular component associated with the largest singular value (μ_2). Colors and shape of nodes indicate the type of the neurons (sensory: red square; interneuron: blue circle; or motor: green diamond). Edge colors indicate the type of the target node with the same color code as for the neuron types. Neuron labels are shown for graph nodes that are projected far from the origin $(0, 0)$ of the axes. We observe that singular value spectrum of wiring network with gap junction is coherent with the one without (gray spectrum). This bimodular embedding is more oriented on the $\mathbf{v}_1 = \mathbf{u}_1$ diagonal which is coherent with the fact that the more a graph is undirected, the more it will be aligned with this main diagonal. We nevertheless observe consistency in the distinction between sensory (red square) and motor (green diamonds) neurons as well as the projection of the *AVE* interneurons on the $\mathbf{u}_1 > 0,\ \mathbf{v}_1 < 0$ quadrant.

SI Dataset S1 (celegans_neurons.csv)

This file summarizes the name, type and physical location of neurons used in this article (original data comes from (1–3)).

References

1. LR Varshney, BL Chen, E Paniagua, DH Hall, DB Chklovskii, Structural Properties of the Caenorhabditis elegans Neuronal Network. *PLOS Comput. Biol.* **7**, e1001066 (2011) Publisher: Public Library of Science.
2. SJ Cook, et al., Whole-animal connectomes of both Caenorhabditis elegans sexes. *Nature* **571**, 63–71 (2019) Publisher: Nature Publishing Group.
3. CA Brittin, SJ Cook, DH Hall, SW Emmons, N Cohen, Volumetric reconstruction of main Caenorhabditis elegans neuropil at two different time points (2018) Pages: 485771 Section: New Results.
